# Hypodopaminergic state of the nigrostriatal pathway drives compulsive alcohol use

**DOI:** 10.1038/s41380-022-01848-5

**Published:** 2022-11-14

**Authors:** Raphaël Goutaudier, Fanny Joly, David Mallet, Magali Bartolomucci, Denis Guicherd, Carole Carcenac, Frédérique Vossier, Thibault Dufourd, Sabrina Boulet, Colin Deransart, Benoit Chovelon, Sebastien Carnicella

**Affiliations:** 1 Inserm, U1216, Univ. Grenoble Alpes, Grenoble Institut Neurosciences, 38000 Grenoble, France; 2 Inserm, U1216, Univ. Grenoble Alpes, CHU Grenoble Alpes, Grenoble Institut Neurosciences, 38000 Grenoble, France; 3 Service de Biochimie, Biologie Moléculaire, Toxicologie Environnementale, CHU de Grenoble-Alpes Site Nord − Institut de Biologie et de Pathologie, F-38041 Grenoble, France; 4 Univ. Grenoble Alpes, CNRS, DPM, 38000 Grenoble, France

**Keywords:** Neuroscience, Addiction

## Abstract

The neurobiological mechanisms underlying compulsive alcohol use, a cardinal feature of alcohol use disorder, remain elusive. The key modulator of motivational processes, dopamine (DA), is suspected to play an important role in this pathology, but its exact role remains to be determined. Here, we found that rats expressing compulsive-like alcohol use, operationalized as punishment-resistant self-administration, showed a decrease in DA levels restricted to the dorsolateral territories of the striatum, the main output structure of the nigrostriatal DA pathway. We then causally demonstrated that chemogenetic-induced selective hypodopaminergia of this pathway resulted in compulsive-like alcohol self-administration in otherwise resilient rats, accompanied by the emergence of alcohol withdrawal-like motivational impairments (i.e., impaired motivation for a natural reinforcer). Finally, the use of the monoamine stabilizer OSU6162, previously reported to correct hypodopaminergic states, transiently decreased compulsive-like alcohol self-administration in vulnerable rats. These results suggest a potential critical role of tonic nigrostriatal hypodopaminergic states in alcohol addiction and provide new insights into our understanding of the neurobiological mechanisms underlying compulsive alcohol use.

## Introduction

More than 300 million people suffer from alcohol use disorder (AUD) worldwide [1], a pathology that encompasses a broad spectrum of health, social and economic problems with various degrees of severity [2]. The most severe form is frequently referred to as alcohol addiction, a chronic relapsing disease occurring in only a subset of vulnerable users [3]. It is notably characterized by compulsive alcohol use, that is the continuation of seeking and drinking alcohol despite having significant negative consequences, and the presence of a negative motivational and affective state in absence of alcohol [2, 4]. While neuroscientists have identified a plethora of actors in AUD, treatments are limited mainly due to the poor understanding of the psychobiological mechanisms underlying the shift from controlled to compulsive alcohol seeking and taking [3]. At the neural system level, dopamine (DA) is considered as a prominent actor in the pathophysiology of addiction, mainly through its role in incentive motivation and reinforcement [4]. However, forty years of research have hitherto failed to provide a clear idea of its exact contribution [5]. While alcohol consumption transiently increases DA levels in the ventral striatum, especially in the nucleus accumbens (NAc), the main output of the mesolimbic system [6], extended consumption instead leads to an overall and prolonged decrease of DA levels upon abstinence in this structure [7–9]. Over the past decade, tonic hypodopaminergic states of the mesolimbic pathway have been causally linked to the emergence of both acute drug withdrawal symptoms and excessive alcohol seeking and taking [7, 9–12]. However, implication of these DA hypofunctions in protracted abstinence and in the compulsive dimension of AUD remains controversial [13, 14].

Beyond the mesolimbic DA pathway, a growing body of research had recently demonstrated the strong implication in motivated and affective behaviors of the neighboring nigrostriatal DA pathway, originally restricted to motor functions [15, 16]. Interestingly, clinical studies in abstinent individuals with AUD have shown an altered DA function not only in the ventral striatum, but also within the dorsal striatum [8, 17], the major output of the nigrostriatal DA pathway, that is assumed to play a prominent role in compulsive drug use [3, 18–20]. More specifically in rodents, compulsive alcohol seeking has been found to depend on the anterior part of the dorsolateral striatum (aDLS) and DA signaling in this structure [21]. In striking contrast with the mesolimbic pathway however, study of potential tonic hypodopaminergic states of the nigrostriatal pathway in AUD has been neglected so far [22]. We therefore hypothesized that the existence of nigrostriatal hypodopaminergia would contribute to the development of compulsive alcohol use and of a negative affective state.

## Materials and methods

### Animals

Experiments were performed on *Wild-Type (WT)* and heterozygous *TH::Cre* Long-Evans male rats. They were housed in a 12 h/12 h reverse light cycle, with food and water *ad libitum*. At the beginning of the experiments, rats were 7 to 8 weeks old. All experimental protocols complied with the European Union 2010 Animal Welfare Act and the new French directive 2010/63, and were approved by the French national ethics committee no. 004.

### Reagents

DREADD agonist C21 (Hello Bio, Bristol, UK) and OSU6162 compound (OSU6162 hydrochloride, Carbosynth Ltd, Compton, UK) were dissolved in 0.9% saline and kept at −20 °C before testing. All the C21 injections were given intraperitoneally at a dose of 0.5 mg/kg (1 ml/kg bodyweight), 1.5 h before the start of the test. For OSU6162 compound, injections were given intraperitoneally at a dose of 7.5 mg/kg or 15 mg/kg (1 ml/kg bodyweight), 1 h before the start of the test [23]. Saline solution (NaCl 0.9%, Sigma, Saint-Quentin-Fallavier, France) was prepared, kept, and injected in identical conditions.

### hM4Di-DREADDs expression

Chemogenetic manipulation of SNc DA neurons was achieved through stereotaxic bilateral infusion of AAV5-hSyn-DIO-hM4D(Gi)-mCherry (10^12^ particles/mL, plasmid #44362, Addgene, Watertown, MA, USA) or AAV5-hSyn-DIO-mCherry (10^12^ particles/mL, plasmid #50459, Addgene) in the SNc of *TH::Cre* rats [24, 25]. As previously described and validated in [24], 1 μL was infused at a rate of 0.2 μL/min into each hemisphere and coordinates for SNc injections were set at: −4.3 mm (AP), ± 2.4 mm (ML), −7.9 mm (DV) relative to bregma [26].

### In vivo microdialysis experiment

Microdialysis experiment was carried out as previously described in [27] and is detailed in Supplement 1. Briefly, homemade microdialysis probes were prepared and bilaterally implanted in the NAc and aDLS. The stereotaxic coordinates were set at: +2.2 mm (AP); ± 3.2 mm (ML) and −6.5 mm (DV) for the aDLS; +2.6 mm (AP); ± 1.2 mm (ML) and −8 mm (DV) for the NAc, relative to bregma [26]. The dialysis fractions were collected every 45 min, over a 6-h period divided in a 1h30 period without treatment and a 4h30 period following injection of C21 or saline solution. After histological validation, DA, 3,4-dihydroxyphenylacetic acid (DOPAC) and homovanillic acid (HVA) contents of dialysis fractions were determined using high-performance liquid chromatography (HPLC).

### Behavioral procedures

#### Intermittent access to 20%-ethanol two-bottle choice drinking procedure (IA 20%-EtOH 2BC)

High levels of voluntary alcohol consumption were obtained using IA 20%-EtOH 2BC drinking procedure as previously described [28, 29]. Briefly, single-housed rats were given 24 h concurrent access to one bottle of 20% (v/v) ethanol (VWR International S.A.S., Fontenay-sous-Bois, France) in tap water and one bottle of water with 24 or 48 h alcohol deprivation periods between the alcohol-drinking sessions. At the end of the procedure, rats consuming less than 2 g/kg/24 h over the last three days were excluded from the study.

#### Operant alcohol self-administration

Rats were trained to orally self-administer alcohol in operant self-administration chambers housed within light-resistant, sound-attenuating boxes (ENV-022MD, Med Associates, St Albans, VT, USA). At the beginning of each session, two levers were extended around the liquid cup: an active, reinforced lever for which presses resulted in the delivery of 0.1 ml of the ethanol solution (20% v/v) in the liquid cup associated to light stimulus above the lever, and an inactive, non-reinforced lever for which a press causes neither delivery of alcohol nor light stimulus. Lever presses on both lever and reinforcer deliveries were recorded by MED-PC IV software (Med Associates). After 2 to 3 nights in the chambers to allow acquisition of a lever-press response for alcohol under a fixed ratio 1 (FR1), operant sessions were conducted 5 days per week, with the schedule requirement increased to FR3 and the length of session shortened from 60 to 30 min over the first 2 weeks [29]. Rats pressing for less than 0.3 g⁄kg⁄30 min at the end of training were excluded from the study.

#### Footshock-punished alcohol self-administration

Identification of rats expressing compulsive-like alcohol use, defined by the persistence of alcohol use despite knowledge of negative associated consequences [3], was achieved by coupling alcohol self-administration with intermittent footshocks. During punished sessions, self-administration parameters were identical to baseline self-administration (i.e., FR3, 30 min) but footshocks (0.25 mA, 0.5 s) were delivered every 8 presses on the reinforced lever, following the paradigm developed by [30]. By doing so, footshock punishment coincide with the preparatory response (lever presses) but not consummatory behaviors (alcohol delivery), promoting punishment of seeking rather than taking [21, 31]. Rats that continued to press the lever, despite footshocks (≥ 70% of the baseline performances), were considered as footshock-resistant whereas rats that stopped pressing the lever in face of the negative consequence were considered as footshock-sensitive. This cut-off was determined based on the variances of the basal operant performances we standardly obtained as well as on the bimodal distribution of our rats’ population (Supplementary Fig. S1A in Supplement1) and confirmed with the Hartigan’s method of K-means clustering (see Supplementary Fig. S2 in Supplement 1).

#### Sensitivity to footshock

Rats were placed in a StartFear chamber (Panlab, Barcelona, Spain) and footshocks were delivered at 0.2, 0.25 and 0.3 mA for experiment 3 (to test whether there was any decrease in footshock sensitivity) and at 0.1, 0.15, 0.2 and 0.25 mA for experiment 5 (to test whether there was any increase in footshock sensitivity). For each intensity, triplicates were made with 20 s intervals, and 1 min was left before increasing the intensity. The footshock threshold was defined as a twitching, a freeze, or a jump off the grid. Footshock responses were recorded with a video-tracking system (EthoVision XT 15 software, Noldus Information Technology) and scored by two observers blind to the experimental conditions.

#### Operant sucrose self-administration

Rats were trained to orally self-administer a 2.5% sucrose (Sigma, Saint-Quentin-Fallavier, France) solution as described for the operant alcohol self-administration, except that each lever press resulted in the delivery of 0.2 mL of sucrose [15]. After 2 to 3 nights in the chambers to allow acquisition of a lever-press response for sucrose under a fixed ratio 1 (FR1), 60 min operant sessions were daily conducted with a maximum of sucrose deliveries set at 100 before ending the session.

#### 2.5% sucrose two-bottle choice drinking test, open arena test, stepping test, light/dark avoidance test, elevated plus-maze and forced-swim test

They were performed according to [15]. A fully detailed description of the procedures is provided for each behavioral test in Supplement 1.

### Striatal tissue DA quantification

aDLS, dorsomedial striatum (DMS) and NAc were dissected from frozen brains and homogenized in: HCl 0.01 N, EDTA 1 mM and Na_2_S_2_O_5_ 4 mM to prevent catecholamine degradation. DA levels were next measured using DA ELISA kit (Immusmol SAS, Talence, France) according to the manufacturer’s instruction and the literature [32–34]. At the end of the reaction, a 0.25 M sulfuric acid was added, and the optical density was detected by spectrophotomety (PHERAstar, BMG Labtech, Champigny sur Marne, France) at the wavelength of 450 nm. A reference wavelength was used at 620 nm to evaluate non-specific absorbance. Finally, DA levels were determined by comparing absorbance of samples with external standards and were expressed as the amount of DA, in ng per mg of protein present in the homogenate determined with BCA Protein Assay Kit (Thermo Scientific, Illkirch, France). Percent of cross-reactivity between DA and its metabolites DOPAC and HVA are < 0.007% (manufacturer provided data). In a first validation step, we verified that the DA ELISA kit provided similar results than HPLC as well as the linearity and the limit of detection of the kit, by testing samples with different dilution with both methods (data not shown).

### Histological analysis

#### Brain tissue preparation and processing

A detailed description of brain tissue preparation and processing is provided in Supplement 1.

#### Quantification of transgene expression

To assess transgene expression in DA mesencephalic regions, immunostaining for tyrosine hydroxylase (TH) was carried out as previously described [24] and as detailed in Supplement 1. DREADD expression was quantified for each hemisphere by comparing the number of TH-labeled-mCherry-positive neurons with the number of TH-labeled neurons within three areas: the distal SNc, the medial SNc and the ventral tegmental area. Rats with less than 30% of transgene expression in the distal SNc in each hemisphere were excluded from the study [24].

### Statistical analyses

Statistical analyses are described in figure legends, summarized in Table S1 and fully detailed in Supplement 1.

### Experimental design

#### Experiment 1

21 *WT* rats were single-housed and trained during 21 sessions in IA 20%-EtOH 2BC. Operant alcohol self-administration was then performed during 38 sessions before the beginning of the footshock-punished alcohol self-administration. At this step, half of the rats performed 6 punished sessions followed by 6 unpunished sessions, while tests were carried out in opposite orders for the other half, thereby ensuring that any decrease in performance was the result of footshock and not an effect of the time or environmental conditions. Additionally, 8 *WT* rats were kept under identical conditions but never exposed to alcohol. To investigate potential long-lasting changes in DA level in 3 striatal sub-regions (NAc, DMS and aDLS), in every experimental subject and at the same time, whole tissue analysis was performed. Rats were therefore euthanized after a period of abstinence (one week after the last self-administration session), and brains were processed for striatal tissues DA quantification with DA ELISA. During striatal tissue quantification, DA levels of each sub-region were analyzed for all rats, except for one rat where technical reasons prevented us from measuring DA levels in DMS.

#### Experiment 2

27 *TH::Cre* rats, housed two to four per cage, were infused with a virus coding for the inhibitory DREADD (designer receptor exclusively activated by designer drug [35]) hM4Di-mCherry or mCherry alone in the substantia nigra pars compacta (SNc). At least two weeks after surgery, allowing the proper recovery of the animals and stable transgene expression [24], microdialysis experiments were performed to investigate the kinetic of the DREADD inhibition. At the end of the experiment, rats were euthanized, and brains were processed for histological validation of the probe placement and transgene expression.

#### Experiment 3

As for experiment 1, 25 *TH::Cre* rats were single-housed and trained in IA 20%-EtOH 2BC and operant alcohol self-administration. After 38 sessions of operant alcohol self-administration, viral surgeries were performed. Following two weeks of recovery, hM4Di and mCherry rats were trained for 8 additional sessions, allowing stabilization of performances, before starting footshock-punished alcohol self-administration. At this step, 5 punished sessions under saline treatment were conducted to identify rats without compulsive alcohol-related behaviors (17 rats), followed by 6 unpunished sessions in absence of treatment (wash period) and, finally, 6 punished sessions under DREADDs agonist Compound 21 (C21) treatment. At the end of the experimental procedure, the sensitivity to footshock was measured. Rats were then euthanized, and brains were processed for histological validation of transgene expression.

#### Experiment 4

48 *TH::Cre* rats, housed two to four per cage, were trained in operant sucrose self-administration during 15 sessions before viral surgeries. After 14 days of recovery, hM4Di and mCherry rats were trained again during 5 sessions, allowing stabilization of performances (≤ 30% performance variation over three consecutive sessions). They were then tested for 3 sessions under C21 or saline treatment. Finally, recovery from treatment (wash period) was assessed during 6 sessions. Following the sucrose self-administration experiment, rats were submitted to, in order: 2.5% sucrose two-bottle choice drinking test, open arena test, stepping test, light/dark avoidance test, elevated plus-maze and forced-swim test. Among the 48 rats, 11 rats that did not reach a minimum of 40 rewards during a session were excluded from this protocol but kept for the rest of the tests. At the end of the experimental procedure, rats were euthanized and brains were processed for histological validation of transgene expression.

#### Experiment 5

*As for experiments 1 and 3, 78 WT* rats were single-housed and trained in IA 20%-EtOH 2BC and operant alcohol self-administration. After 40 sessions of operant alcohol self-administration, footshock-punished alcohol self-administration was started. At this step, 6 punished sessions were conducted to identify rats with compulsive-like alcohol use (28 rats), followed by 15 unpunished sessions in absence of treatment and, 6 punished sessions under saline or OSU6162 (7.5 mg/kg) treatments. Rats then underwent 10 unpunished sessions in absence of treatment, allowing recovery and stabilization of operant performances and were again tested during 6 punished sessions under saline or OSU6162 (15 mg/kg). On the 28 “compulsive-like” rats, two animals from the saline condition were later excluded from the data for abnormal decrease in operant performances during unpunished self-administration. At the end of the experimental procedure, the sensitivity to footshock was measured. The effect of each dose of OSU on general operant behavior was also assessed during unpunished alcohol self-administration.

## Results

### Compulsive alcohol use is specifically associated with a decrease in DA levels in the aDLS

**Fig. 1:**
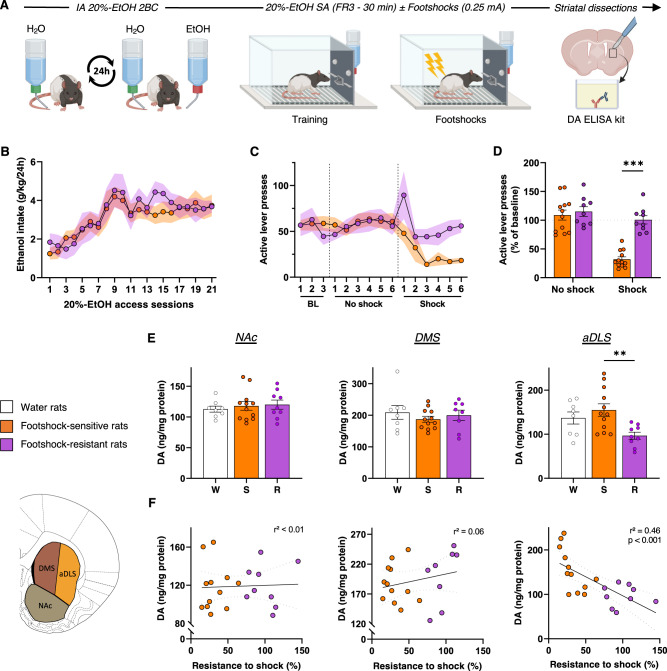
Compulsive alcohol use is specifically associated with a decrease in DA levels in the aDLS. **A** Experiment timeline. **B** Alcohol intake during intermittent-access 20%-ethanol two-bottle-choice (*IA 20%-EtOH 2BC*). RM two-way ANOVA showed a significant effect of session [F_(6, 109)_ = 7.82, *P* < 0.001, partial η^2^ = 0.29] but neither effect of group, nor session x group interaction [F_s_ < 0.47, *P* > 0.5, partial η^2^ < 0.02]. **C** Number of active lever presses in 30-min self-administration sessions (SA) of 20% EtOH (FR3), during baseline (BL), “no-shock” and “shock” sessions (see supplementary material for details) in *footshock-sensitive* (FS, orange, *n* = 12) and *footshock-resistant* (FR, purple, *n* = 9) rats. RM two-way ANOVA showed a significant group x session interaction [F_(14, 266)_ = 2.86, *P* < 0.001, partial η^2^ = 0.13]. **D** Mean active lever presses during the last three “no-shock” sessions and the last three “shock” sessions normalized to baseline. RM two-way ANOVA showed a significant shock condition x group interaction [F_(1, 19)_ = 20.89, *P* < 0.001, partial η^2^ = 0.52]. **E** NAc, DMS and aDLS DA levels for *FR*, *FS* and *Water* rats (white, *n* = 8). Mixed-effects analysis found a group x striatal sub-regio*n* interaction [F_(4, 77)_ = 2.96, *P* < 0.05]. **F** Linear regression between resistance to footshock and DA level in NAc, DMS or aDLS. A significant correlation was found in the aDLS [F_(1,19)_ = 16.18, *P* < 0.001], but not in the NAc or DMS [F_s_ < 1.14, *P* > 0.3]. Data are expressed as mean ± SEM. Bonferroni correction post-hoc analysis: **, *P* < 0.01; ***, *P* < 0.001. BL baseline, DMS dorsomedial striatum, aDLS anterior dorsolateral striatum, FR fixed ratio, NAc nucleus accumbens.

We first tested whether compulsive alcohol use is associated with a hypodopaminergic state of the nigrostriatal pathway, by assessing striatal DA levels in rats with or without compulsive-like alcohol seeking behavior. In the first experiment, animals were first trained to voluntarily consume alcohol in IA 20%-EtOH 2BC, before being challenged instrumentally to respond to alcohol in operant chambers [28, 29]. When operant performances reached stable levels, lever presses for alcohol were coupled to mild footshocks to identify rats with compulsive-like alcohol use, operationalized as punishment-resistant alcohol self-administration [30, 36] (Fig. 1A). During these punished sessions, a bimodal distribution progressively appeared, clearly indicating the existence of two distinct sub-populations: one with a strong decrease in lever presses, the footshock-sensitive (FS) rats (58%), while the other, the footshock-resistant (FR) rats (42%), maintained their responses despite footshocks (Fig. 1C, D, Supplementary Fig. S1A in Supplement 1; see also [36, 37]). No differences were observed on the second, inactive lever, indicating that the tendency to resist to punishment was specifically related to the search for alcohol and not to a general increase in lever response activity (Supplementary Fig. S1B in Supplement 1). Importantly, and as already observed for alcohol [21] and cocaine [38], the history of intake cannot account for these two distinct phenotypes. Indeed, we found a similar escalation of alcohol consumption during IA 20%-EtOH 2BC (Fig. 1B and Supplementary Fig. S1C in Supplement 1), as well as similar lever presses during unpunished self-administration sessions between FR and FS rats (Fig. 1C, D). While DA levels measured by ELISA assays on striatal extracts did not differ between FS and FR rats in the NAc or DMS and appeared similar to those obtained without alcohol exposition (water control rats), FR rats showed a significant decrease in DA levels in the aDLS (Fig. 1E). In addition, a robust negative correlation was found between the degree of resistance to footshock and the amount of aDLS DA (Fig. 1F). Thus, in line with our working hypothesis, compulsive-like alcohol seeking is associated with a decrease in aDLS DA, suggesting an important link between a tonic hypodopaminergic state of the nigrostriatal pathway and the emergence of this maladaptive behavior.

### Chemogenetically-induced nigrostriatal hypodopaminergia induces compulsive alcohol use

**Fig. 2:**
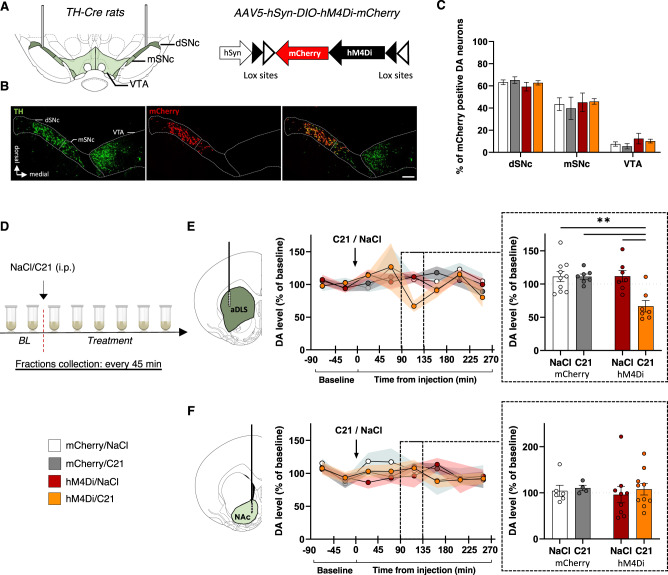
Chemogenetic inhibition of the SNc DA neurons induces selective nigrostriatal hypodopaminergia. **A–C** hM4Di-mCherry and mCherry expression in SNc DA neurons. **A** *TH::Cre* rats received bilateral injection of Cre-dependent hM4Di-mCherry or Cre-dependent mCherry virus in the SNc. **B** Representative images of TH immunostaining and hM4Di-mCherry expression. Scale bar: 250 μm. **C** Quantification of transgene expression in distal (dSNc), medial SNc (mSNc) and VTA. Three-way ANOVA showed a significant effect of the area [F_(2,129)_ = 158, *P* < 0.001, η^2^ = 0.71], but no effect of transgene, treatment or any interaction between these factors [F_s_ < 1.04, *P* > 0.36, partial η^2^ < 0.02]. **D–G** Extracellular DA concentrations in aDLS and NAc throughout eight 45 min-fractions collected by microdialysis. Data are normalized to baseline. **D** Design of the microdialysis experiment. **E** Time course of extracellular DA concentrations in the aDLS of *hM4Di* rats treated with C21 (orange, *n* = 7) or NaCl (red, *n* = 7) and *mCherry* rats treated with C21 (gray, *n* = 7) or NaCl (white, *n* = 10). **F** Time course of extracellular DA concentrations in the NAc of *hM4Di* rats treated with C21 (orange, *n* = 10) or NaCl (red, *n* = 9) and *mCherry* rats treated with C21 (gray, *n* = 4) or NaCl (white, *n* = 6). In the fractio*n* collected between 90 a*n*d 135 minutes after injection (dotted squares), two-way ANOVA found a treatment x transgene interaction in the aDLS [F_(1,27)_ = 8.63, *P* < 0.01, partial η^2^ = 0.24], but not in the NAc [F_(1,25)_ = 0.03, *P* > 0.5, partial η^2^ = 0.001]. Data are expressed as mean ± SEM. Bonferroni correction post-hoc analysis: **, *P* < 0.01. C21: compound 21, SNc substantia nigra pars compacta, TH tyrosine hydroxylase, VTA ventral tegmental area.

Consequently, we next investigated whether this DA hypofunction is causally implicated in the emergence of compulsive alcohol use. To this end, experimental and reversible nigrostriatal hypodopaminergia was induced using the inhibitory DREADD hM4Di [35]. *TH::Cre* rats were transduced in the SNc with AAVs that Cre-dependently express hM4Di-mCherry, or mCherry alone (control condition), allowing its specific expression in SNc DA neurons (Fig. 2A–C). hM4Di was activated by the synthetic ligand C21 [39], at a dose that we previously reported as potent and selective to inhibit SNc neurons in *TH::Cre* rats [24]. We first confirmed with in vivo microdialysis that this approach efficiently decreased aDLS DA (Fig. 2D and Supplementary Fig. S3 in Supplement 1). In hM4Di rats, a 30%-decrease in aDLS DA was observed between 90 and 135 minutes after C21 injection (Fig. 2E), which is consistent with the temporal inhibition of SNc neurons previously reported [24]. In contrast, the levels of the metabolites DOPAC and HVA were not modified (Supplementary Fig. S4A in Supplement 1), indicating that the decrease in aDLS DA rather reflects lower release than a higher turnover rate, in accordance with the chemogenetic inhibition of SNc DA neurons. Importantly, this strategy influenced neither DA nor DOPAC and HVA levels in the NAc (Fig. 2E and Supplementary Fig. S4B in Supplement 1), confirming that the reversible chemogenetic induction of a hypodopaminergic state is selective of the nigrostriatal pathway.

**Fig. 3:**
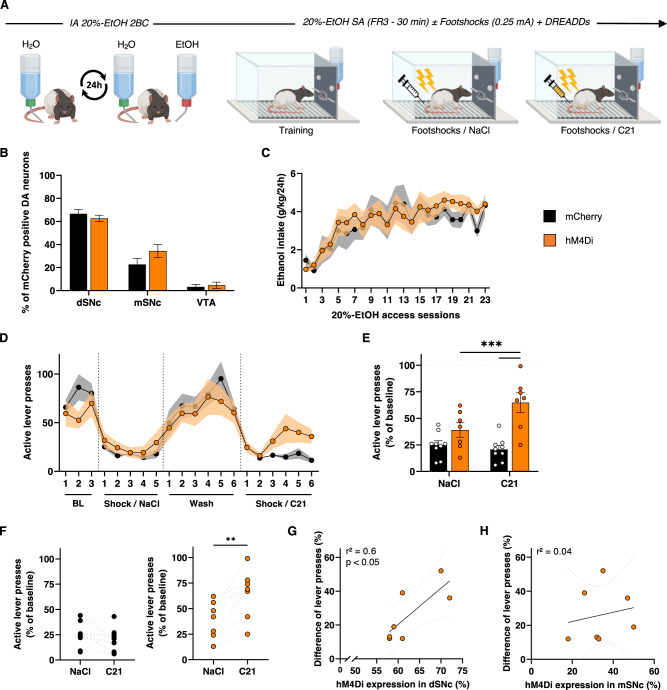
Chemogenetically-induced nigrostriatal hypodopaminergia induces compulsive alcohol use. **A** Experiment timeline. **B** Quantification of hM4Di-mCherry and mCherry expression in distal (dSNc), medial SNc (mSNc) and VTA. Two-way ANOVA showed significant effect of the area [F_(2,90)_ = 116.4, *P* < 0.001, η^2^ = 0.72], but no effect of transgene, treatment or any interaction between these factors [F_s_ < 1.99, *P* > 0.14, partial η^2^ < 0.04]. **C** Alcohol intake during *IA2BC 20%-EtOH* in *hM4Di* rats (orange, *n* = 7) and *mCherry* rats (black, *n* = 10). RM two-way ANOVA showed a significant effect of session [F_(4, 59)_ = 10.56], *P* < 0.001, partial (η^2^ = 0.43), but neither effect of transgene, nor session x transgene interaction [F_s_ < 0.57, *P* > 0.5, partial η^2^ < 0.04]. **D** Number of active lever presses in 30-min self-administration sessions of 20%-EtOH **(**FR3), during baseline (BL), “Shock/NaCl”, “Wash” and “Shock/C21” sessions. RM two-way ANOVA showed a significant session x transgene interaction [F_(19,266)_ = 2.18, *P* < 0.01, partial η^2^ = 0.13]. Mean active lever presses during the last three “Shock/NaCl” and “Shock/C21” sessions normalized to baseline (**E**) and individual trajectories during these two periods (**F**). RM two-way ANOVA reported a significant session x transgene interaction [F_(1,14)_ = 18.41, *P* < 0.001, partial η^2^ = 0.57]. Correlation between the difference of active lever presses during “Shock/C21” and “Shock/NaCl” sessions and the percent of hM4Di expression within dSNc (**G**) or mSNc (**H**). A significant correlation was found for dSNc [F_(1,5)_ = 7.64, *P* < 0.05], but not for mSNc [F_(1,5)_ = 0.18, *P* > 0.5]. Data are expressed as mean ± SEM. Bonferroni correction post-hoc analysis: **, *P* < 0.01; ***, *P* < 0.001.

We next tested whether induction of this experimental nigrostriatal hypodopaminergia is sufficient to induce compulsive-like alcohol seeking in FS rats. In this third experiment, *TH::Cre* rats were therefore trained in IA 20%-EtOH 2BC and operant alcohol self-administration procedures as in the first experiment (Fig. 3A), and then transduced with DREADDs ensuring the same history of alcohol exposure between hM4Di and mCherry control rats (Fig. 3B and C). After selection of FS-hM4Di and FS-mCherry rats under punishment sessions preceded by saline injections, we assessed their tendency to persist in seeking alcohol despite punishment over the course of a second series of sessions, this time following administration of C21 (Fig. 3D). In these sessions, we found that chemogenetic inhibition of SNc DA neurons progressively increased the resistance of FS rats to self-administer the drug under punishment (Fig. 3D and E), while it did not affect their intrinsic footshock sensitivity (Supplementary Fig. S5A in Supplement 1). Interestingly, this increase was observed for each hM4Di FS rat (Fig. 3F) and correlated only with the level of hM4Di expression within the distal SNc (Fig. 3G, H), the main DA input of the DLS [40]. In marked contrast, chemogenetic manipulation had no effect on either the inactive lever presses during punishment sessions (Supplementary Fig. S5B in Supplement 1), or on operant alcohol self-administration under baseline conditions (Supplementary Fig. S5C in Supplement 1), confirming that this effect was related to punishment and not to a non-selective or general change in operant behavior. Taken together, these results demonstrated that an hypodopaminergic state of the nigrostriatal pathway is sufficient to induce compulsive-like alcohol seeking behavior in animals that were otherwise resilient.

### Chemogenetically-induced nigrostriatal hypodopaminergia leads to a prolonged negative motivational, but not affective, state

**Fig. 4:**
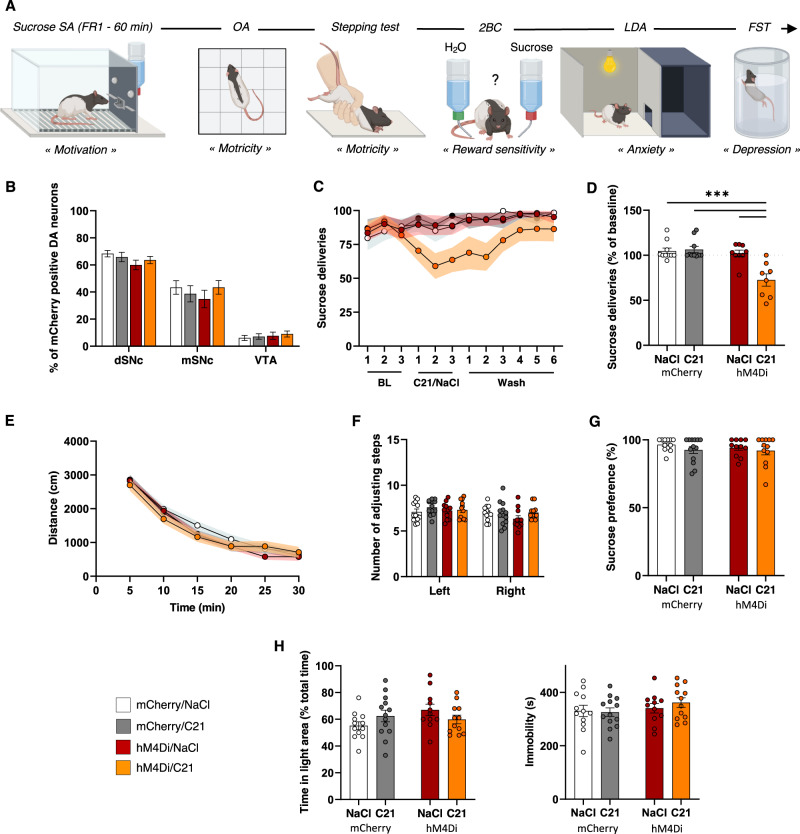
Chemogenetically-induced nigrostriatal hypodopaminergia leads to a prolonged negative motivational, but not affective, state. **A** Behavioral screening timeline. **B** Quantification of hM4Di-mCherry and mCherry expression in distal (dSNc), medial SNc (mSNc) and VTA. Three-way ANOVA showed significant effect of the area [F_(2, 140)_ = 346.7, *P* < 0.01, η^2^ = 0.83], but no effect of transgene, treatment or any interaction between these factors [F_s_ < 1.31, *P* > 0.27, partial η^2^ < 0.02]. **C** Number of 2.5%-sucrose deliveries obtained in 60-min self-administration (SA) sessions under continuous reinforcement (FR1), during baseline (BL), “C21/NaCl” and “Wash” sessions, in *hM4Di* rats treated with C21 (orange, *n* = 8) or NaCl (red, *n* = 9) and *mCherry* rats treated with C21 (gray, *n* = 10) or NaCl (white, *n* = 10). RM three-way ANOVA showed a significant session x transgene x treatment interaction [F_(11, 363)_ = 2.67, *P* < 0.01, partial η^2^ = 0.07]. **D** Mean sucrose deliveries obtained during “C21/NaCl” sessions normalized to baseline. Two-way ANOVA showed a significant transgene x treatment interaction [F_(1, 33)_ = 13.38, *P* < 0.001, partial η^2^ = 0.29]. **E**—**I** Distance traveled over the course of a 30-min session in an open area (OA) (E), number of adjusting left and right forepaws in a stepping test (**F**), sucrose preference measured over a 60-min 2.5%-sucrose two-bottle**-**choice (2BC) drinking session (**G**), percentage of time spent in the light area in a light/dark avoidance test (LDA) (**H**) and time spent immobile in a forced swim test (FST) (I) in *hM4Di* rats treated with C21 (orange, *n* = 12) or NaCl (red, *n* = 11) and *mCherry* rats treated with C21 (gray, *n* = 13) or NaCl (white, *n* = 12). Two- or three-way ANOVA found no interaction implicating the transgene and the treatment [F_s_ < 1.7, *P* > 0.2, partial η^2^ < 0.07] in these different tests, except a marginal side x transgene x treatment interaction in stepping test [F_(1, 44)_ = 3.55, *P* = 0.07, partial η^2^ = 0.07] and a transgene x treatment interaction in LDA [F_(1, 44)_ = 3.68, *P* = 0.06, partial η^2^ = 0.08], mainly driven by small size effects related to the transgene condition and not to a C21 effect on *hM4Di* rats. Data are expressed as mean ± SEM. Bonferroni correction post-hoc analysis: ***, *P* < 0.001.

Because we previously showed that partial DA denervation of the nigrostriatal pathway leads to motivational impairments as well as depression- and anxiety-like behaviors [15], three core features of the negative psychological state experienced during withdrawal [41], we finally test whether chemogenetically-induced nigrostriatal hypodopaminergia reproduced this phenotype (Fig. 4A, B). Motivated behavior was evaluated in operant sucrose self-administration task under a fixed and progressive ratio schedule of reinforcement as previously performed [15, 16]. In comparison to the control groups, hM4Di-C21 treated rats exhibited a prolonged decrease in their performance to obtain sucrose, that persisted beyond the last session under C21 (Fig. 4C, D and Supplementary Fig. S6A in Supplement 1). This result was not due to a motor deficit associated with nigrostriatal hypodopaminergia, as revealed by the absence of motor alterations in an open arena and fine use of the forepaws in a stepping test (Fig. 4E, F). It cannot be attributed to an inability to discriminate between the active and inactive levers that was preserved throughout the task (Supplementary Fig. S6B in Supplement 1), or to a decrease in sensitivity to the reinforcing properties of sucrose, as preference (Fig. 4G) and consumption (Supplementary Fig. S6C, in Supplement 1) for the sucrose solution was unchanged in the two-bottle choice paradigm. In addition, general consummatory behaviors were not affected, as animal body weights were not altered by the chemogenetic manipulation through the administration period (data not shown). Taken together, these data confirm that the chemogenetically-induced decrease in operant sucrose behavior reflects a motivational deficit [15]. In sharp contrast, anxiety- or depression-like behaviors were not increase in light/dark avoidance and elevated-plus maze tests (Fig. 4H and Supplementary Fig. S6D in Supplement 1) and in the forced swim test (Fig. 4I) respectively. Although immobility levels were higher than previously reported (e.g., [15, 42]), percentages of immobility remain between 54% and 60% of the total time of the experiment making the absence of differences unlikely to be explained by a potential ceiling effect. Finally, in addition to its implication in compulsive alcohol use, the hypodopaminergic state of the nigrostriatal pathway leads to a prolonged negative motivational, but not affective, state.

### The monoamine stabilizer OSU6162 temporarily reduces compulsive alcohol use

**Fig. 5:**
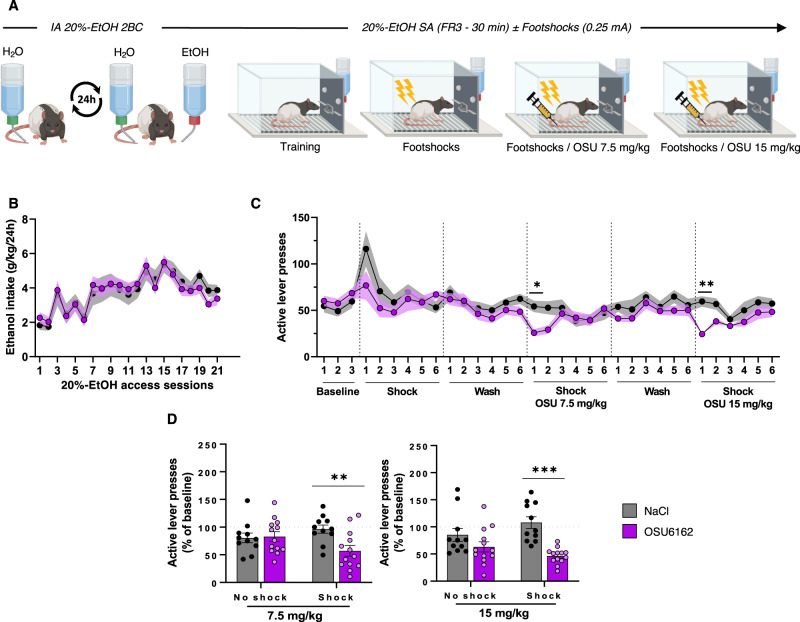
OSU6162 compound temporarily decreased compulsive behavior in footshock-resistant rats. **A** Experiment timeline. **B** Alcohol intake during IA 20%-EtOH 2BC in *NaCl* rats (black, *n* = 12) and *OSU* rats (purple, *n* = 14). RM two-way ANOVA showed a significant effect of session [F_(4, 115)_ = 18.4, *P* < 0.001, partial η^2^ = 0.43] but no effect of group or session x group interaction [F_s_ < 0.75, *P* > 0.5, partial η^2^ < 0.03]. **C** Number of active lever presses in 30-min self-administration sessions of 20%-EtOH (FR3), during baseline, “Shock”, “Wash” and “Shock/OSU” sessions at either 7.5 mg/kg or 15 mg/kg. Mixed-effects analysis showed a significant session x treatment interaction [F_(32, 761)_ = 1.6, *P* < 0.05]. **D** Mean active lever presses during the “No shock” and the first “Shock” session with OSU administration at 7.5 mg/kg (left) or 15 mg/kg (right) normalized to baseline. For both conditions, RM two-way ANOVA showed a significant effect of the treatment [F_s_ > 7.17; *P* < 0.05; η^2^ > 0.13] with a marginal treatment x shock interaction [F_s_ > 3.57; *P* < 0.07; η^2^ > 0.14] but no effect of the shock [F_s_ < 0.24; *P* > 0.5; η^2^ < 0.01]. Data are expressed as mean ± SEM. Bonferroni correction post-hoc analysis: *, *P* < 0.05; **, *P* < 0.01; ***, *P* < 0.001.

As we demonstrated that the hypodopaminergic state of the nigrostriatal pathway is involved in the development of compulsive alcohol-related behavior, we hypothesized that normalization of DA tone could relieve this maladaptive behavior. To test this, *WT* rats were trained in IA 20%-EtOH 2BC and operant alcohol-self-administration procedures as in the previous experiments (Fig. 5A, B). After selection of FR rats under punished sessions, we sub-chronically administered the monoamine stabilizer OSU6162, a partial agonist of D2-R and 5-HT2A-R [43, 44] that was previously reported to normalize DA tone [23, 45]. Whatever the dose used, we observed that OSU6162 treatment significantly and transiently decreased the resistance to self-administer the drug under punishment, from the first punished session before returning to baseline the 3^rd^ session (Fig. 5C, D). Importantly, this effect was strictly related to drug seeking under punishment and not to a non-selective or general change in activity or operant behavior, as OSU6162 treatment did not significantly alter unpunished alcohol self-administration (Fig. 5D). It was also not due to an alteration in lever discrimination, as inactive lever responses did not increase (Supplementary Fig. S7A in Supplement 1). In addition, intrinsic footshock sensitivity were not affected by the pharmacological treatment (Supplementary Fig. S7B in Supplement 1). Taken together, these results indicate that the monoamine stabilizer OSU6162 can specifically reduce compulsive-like alcohol seeking behavior in rats that are otherwise resistant.

## Discussion

Despite a long history of research on DA, its exact role in addiction remains a matter of debate [5, 14]. Here, we shed to light a potential implication of the DA nigrostriatal pathway in AUD. Our findings indicate that chronic exposure to alcohol leads to the development of a tonic hypodopaminergic state in the nigrostriatal pathway in vulnerable individuals that directly contributes to the development of compulsive alcohol use and may participates to the maintenance of the negative motivational state experienced during abstinence. Our study does not however preclude potential involvement of the mesolimbic pathway in compulsive alcohol use. Indeed, the investigation of DA level within whole tissues for experimental reasons (see experimental design) may have masked a potential alteration limited to the extracellular level in the NAc or the DMS. Moreover, potential subregional effects between NAc core and shell may have been missed, even though previous studies have reported alcohol-induced hypodopaminergia in the NAc without dissociating these two sub-structures (e.g. [9]). Nevertheless, our results suggest that these potential alterations are much less profound than those observed in the DLS, since DA alteration was found at the tissular level, at least after one week of alcohol withdrawal, implicating multiple and strong neuroadaptations within the DA system [46–49].

Mesolimbic tonic hypodopaminergic states have been proposed to be part of the allostasis phenomenon observed during abstinence that underlies the emergence of a negative motivational and affective state and participates in the development of excessive alcohol seeking and intake [9, 11, 12, 50]. The present data suggest that, in vulnerable chronic users, these DA hypofunctions might eventually propagate or transfer to the nigrostriatal pathway through different possible interconnexions [51, 52]. This might be the tonic counterpart of striatal DA phasic signal transition, that have been observed to emerge progressively in the aDLS over the course of drug taking, while concomitantly fading in the ventral portion [53]. Interestingly, DA phasic signals are directly modulated by DA tonic state [54] and the relation between these two complementary modes of signaling has been suggested to be important for alcohol and other substance use disorder [55]. Emergence of the hypodopaminergic state of the mesolimbic pathway in the early phase of the disorder might therefore lead to aberrant enhanced DA phasic signaling in the NAc, that would contribute to aberrant learning [56] and sensitization of the mesolimbic DA pathway to drugs or associated cue [57, 58]. Within this framework, complex interplay between DA tone and DA phasic signals has already been reported using voltametric and microdialysis approaches, where chronic substance use persistently decreased DA tone while, in parallel, increased DA phasic responses to salient stimuli, thereby increasing “signal-to-noise” ratio in the NAc [59–62]. In addition, optogenetic studies conducted within the mesolimbic pathway have shown that increasing DA phasic firing promotes excessive alcohol seeking, whereas increasing DA tonic firing decreases consumption and abolishes the effect of DA phasic signal when induced simultaneously [12, 63, 64]. In the ultimate stages of AUD, the hypodopaminergic state of the nigrostriatal pathway revealed in our study, would strengthen similar abnormal phasic DA signals in the aDLS engaged by drug-paired cues, facilitating the formation of maladaptive incentive habits and the ensuing rigidity in drug seeking [21, 52, 65] exacerbated by negative urgency during abstinence [66].

Additionally, a DA deficit in the nigrostriatal pathway may reduce the capacity to learn or to use (because in the chemogenetic experiment, rats had learned well the relation between responding on the lever and the footshock before the induction of the hypodopaminergic state) the aversive information carried by the footshock to inhibit operant responding (see also [67]). Indeed, the nigrostriatal DA pathway has been shown to signal aversive events [68, 69] and the dorsal striatum to play an important role in punishment-based avoidance learning in humans [70].

These hypotheses therefore suggest that DA-based therapy normalizing DA tonic signaling would suppress excessive as well as compulsive alcohol seeking and drinking. In this respect, normalization of DA tone in the mesolimbic pathway, using GDNF or OSU6162, has already been shown to suppress excessive alcohol consumption in rodents and diminish alcohol craving in humans [9, 23, 45]. Regarding the more severe form of AUD, this therapeutic strategy on compulsive alcohol use has never been specifically assessed. Here we demonstrate that the monoamine stabilizer OSU6162, known to correct DA deficiencies [23, 45], transiently reduces compulsive-like alcohol use. This transient effect may suggest compensatory, or tolerance, mechanisms in response to the repeated exposure to the molecule and/or may be due to the use of low to moderate doses here. Higher doses have not been tested here as they have been shown to efficiently reduce alcohol intake in rats in absence of punishment, while the objective was to specifically investigate potential effects of OSU6162 on compulsivity [23, 71]. From a therapeutic perspective however, the use of a higher dose could be highly relevant to treat the most severe forms of AUD by reducing both alcohol consumption and the compulsive behaviors associated with it. Although the underlying mechanisms remain to be investigated, our results with OSU6162 provide a valuable first proof of concept that a stabilizer of DA function can reduce compulsive alcohol-related behavior, as well as bringing new insight for the development of a promising treatment to sustainably limit compulsive alcohol use.

Although our study was focused on midbrain DA neurons and their projections to the striatum, other structures play an important role in compulsive alcohol use. As such, cortical structures such as the prefrontal cortex (PFC) [67, 72] and orbitofrontal cortex (OFC) [73–75] have been shown to be involved in loss of control, impaired decision making, and compulsive-like behaviors associated with drugs of abuse. It is notably suggested that the transition to compulsion is controlled, in part, by cortico-striatal projections [3, 18, 22]. This hypothesis is supported by studies demonstrating hypoactivity of the mPFC [67, 72] and hyperactivity of OFC projections to the dorsal striatum [76–78], leading to an imbalance between these two structures [79] in mice with compulsive-like behaviors. Taken with the present findings, the emergence of the hypodopaminergic state of the nigrostriatal pathway and the imbalance between mPFC and OFC may act in synergy to drive compulsive drug use. Indeed, through the tripartite synapses between glutamate and dopamine afferents onto medium spiny neurons, DA terminals directly modulate cortico-striatal connectivity [80] and an alteration of the DA tone may thereby contribute to top-down control impairment.

The hypothesis of allostasis proposes that negative affective states during withdrawal participate in the development of compulsive alcohol use [50, 81]. Here, we show that induction of a nigrostriatal hypodopaminergic state led to motivational, but not anxiety- or depression-like behaviors reminiscent of withdrawal. Greater DA deficiency of the nigrostriatal pathway may be necessary for a negative affective state to occur [15] or may be associated with DA deficiencies in the mesolimbic pathway or other neurotransmitter systems as previously evidenced [4, 81]. In addition, although the alcohol drinking and self-administration procedure used in the present study is highly relevant in respect to excessive alcohol use as well as compulsive-like behaviors, and induces some signs of physical and psychological withdrawal ([28, 30] and see Supplementary Methods in Supplement 1), it does not model alcohol dependence as other models [28], such as vapor chambers [82, 83]. Therefore, we highlight here the causal implication of hypodopaminergic states in the development of compulsive alcohol use, but [81, 82] investigations using strictly designed models of alcohol dependence will be required to deeply investigate the causal relationship with withdrawal-related negative affective states.

Beside compulsivity and withdrawal states, our study also intriguingly reports a dissimilar effect of nigrostriatal hypodopaminergia on instrumental behaviors to obtain alcohol and sucrose. Indeed, whereas nigrostriatal hypodopaminergia did not change alcohol self-administration, and even increased resilience to obtain alcohol in the presence of footshock, it markedly decreased motivation to self-administer sucrose. This suggests that the hypodopaminergic state of the nigrostriatal pathway may have different behavioral outcomes depending on the natural properties of the reward and its associated history. If this observation requires further investigations to directly compare motivation for sucrose and alcohol [84, 85], it represents an interesting feature to note, as a shift in interest in the drug at the expense of other natural rewards or activities is an important dimension of addiction [2, 86, 87]. Interestingly, Augier et al. has already demonstrated that rodents preferring alcohol over saccharin have GABAergic hyperactivity in the central amygdala [85], a structure involved in compulsive alcohol use [36] and that also projects to the SNc [88, 89].

In conclusion, this study points towards the causal implication of a tonic hypodopaminergic state of the nigrostriatal pathway in compulsive alcohol use, a cardinal feature of AUD [3]. These data also emphasize the importance of the nigrostriatal DA pathway in motivated behavior and suggest that this hypodopaminergic state may contribute to the negative motivational state observed during withdrawal. The role of this pathway in motivation-related processes has long been neglected [15], while it could represent one of the key elements to understanding the complex physiopathology of AUD. Future work will be necessary to decipher the neurobiological and molecular mechanisms underlying this tonic hypodopaminergic state and how it leads to the emergence of compulsive alcohol use. In addition, it remains to be determined whether this mechanism is specific to alcohol or common to all drugs of abuse, as DA alteration has already been found in the DS for cocaine for instance [90, 91]. Finally, this study was conducted only in male rats as we reported that C21 may have had potential off-target effects in females that mitigated the induction of the hypodopaminergic state [24]. As important sex differences have been reported regarding drug-related behaviors, drug-induced neuroadaptations, or DA systems [92–94], it will be crucial to extend this study to female. Although these questions remain, our results open new avenues of research in AUD and therapeutic strategies based on the restoration of normal DA tone to abolish this maladaptive behavior.

## Supplementary information

Table S1

Supplemental Material 1
