# Supplementary material for: Hypodopaminergic state of the nigrostriatal pathway drives compulsive alcohol use
Source: Mol Psychiatry. 2022 Nov 14;28(1):463–74. doi: 10.1038/s41380-022-01848-5 (PMC9812783; doi:10.1038/s41380-022-01848-5)
Supplement: Supplementary file 2 — Supplemental Material 1 [file 41380_2022_1848_MOESM2_ESM.pdf]

SUPPLEMENT 1

Hypodopaminergic state of the nigrostriatal pathway drives compulsive alcohol use

Running title: Nigrostriatal hypodopaminergia drives compulsivity

Authors: Raphaël Goutaudier Ph.D[1#], Fanny Joly Ph.D[1#], David Mallet Ph.D[1], Magali Bartolomucci M.S[2], Denis Guicherd B.S[3], Carole Carcenac Ph.D[1], Frédérique Vossier B.S[1], Thibault Dufourd Ph.D[1], Sabrina Boulet Ph.D[1], Colin Deransart Ph.D[2], Benoit Chovelon Ph.D[3,4], Sebastien Carnicella Ph.D[1*]

Affiliation:
[1]Inserm, U1216, Univ. Grenoble Alpes, Grenoble Institut Neurosciences, 38000 Grenoble, France
[2]Inserm, U1216, Univ. Grenoble Alpes, CHU Grenoble Alpes, Grenoble Institut Neurosciences, 38000 Grenoble, France
[3]Service de Biochimie, Biologie Moléculaire, Toxicologie Environnementale, CHU de Grenoble-Alpes Site Nord − Institut de Biologie et de Pathologie, F-38041 Grenoble, France.
[4]Univ. Grenoble Alpes, CNRS, DPM, 38000, Grenoble, France

[#]Co-first authors

***Corresponding author:**
Sebastien Carnicella, Ph.D.
Grenoble Institute of Neurosciences
31 Chemin Fortuné Ferrini
38700 La Tronche
France
E-mail: sebastien.carnicella@inserm.fr
Tel.: +33-(0)4-56-52-06-67/(0)6-23-88-26-65

***Supplement 1** contains:*

- \- Supplemental Methods
- \- Suppl. Fig. S1 to S4
- \- Suppl. Table S1

SUPPLEMENTAL METHODS

Animals

Rats used in this study were bred at the Grenoble Institute of Neurosciences and the associated Plateforme de Haute Technologie Animale (Experiment 1, 2, 3, 4; La Tronche, France), and the Janvier Labs (genetic background maintenance and Experiment 5; Le Genest-Saint-Isle, France). All experimental protocols complied with the European Union 2010 Animal Welfare Act and the new French directive 2010/63, and were approved by the French national ethics committee no. 004.

hM4Di-DREADDs expression

Chemogenetic manipulation of SNc DA neurons was achieved through stereotaxic bilateral infusion of AAV5-hSyn-DIO-hM4D(Gi)-mCherry (10^{12} particles/mL, plasmid #44362, Addgene, Watertown, MA, USA) or AAV5-hSyn-DIO-mCherry (10^{12} particles/mL, plasmid #50459, Addgene) in the SNc of *TH-Cre* rats (1,2). Animals were anesthetized with a mixed intraperitoneal injection of ketamine (Chlorkétam, 60 mg/kg, Mérial SAS, Lyon, France) and xylazine (Rompun, 10 mg/kg, Bayer Santé, Puteaux, France). Then local anesthesia was provided by a subcutaneous injection of lidocaïne (Lurocaïne, 8 mg/kg, Vetoquinol S.A., Lure, France) on the skull surface and animal were secured in a Kopf stereotaxic frame (Phymep, Paris, France) under a microbiological safety post (PSM). Coordinates for SNc injections were determined according to (3), adjusted to the body weight, and set at: : -4.3 mm (AP), ±2.4 mm (ML), -7.9 mm (DV), relative to bregma. 1 µL was infused into each hemisphere at a rate of 0.2 µL/min using microinjection cannula (33-gauge, Plastic One, USA) connected to a 10 µL Hamilton syringe and a microinjection pump (Stoelting Co., Wood Dale, IL, USA). The cannula was then left in place for 5 min to allow the injected solution to be absorbed into the parenchyma and minimize the spread of the virus along the cannula tract. The skin was sutured, disinfected,

and the animal placed in a heated wake-up cage, before being replaced in its home-cage after complete awakening and monitored for several days. With these parameters, we always observed mCherry staining in TH-positive neurons. This selective expression of the transgene was confirmed in a pilot experiment in which no mCherry staining was detected after the total destruction of SNc DA neurons with the neurotoxin 6-hydroxydopamine (data not shown).

***In vivo* microdialysis experiment**

Rats were anesthetized by inhalation of isoflurane (Isoflurin, Axience SAS, Pantin, France) and secured in a Kopf stereotaxic frame (Phymep, Paris, France). Low concentrations of isoflurane were used (< 2%) to avoid alteration of DA signaling (4). The dorsal skull was exposed, and holes were drilled to facilitate the bilateral implantation of microdialysis probes. Homemade microdialysis probes were prepared and the length of the dialysis membrane was adapted to the brain regions studied (1.8 mm for the anterior dorsolateral striatum (aDLS) and 1 mm for the nucleus accumbens (NAc)). During each experiment, one probe was lowered in the aDLS on one side and one probe was lowered in the NAc on the other side. The stereotaxic coordinates relative to bregma were determined according to (3), adjusted to the body weight, and set at: +2.2 mm (AP); ±3.2 mm (ML) and -6.5 mm (DV) for the aDLS; +2.6 mm (AP); ±1.2 mm (ML) and -8 mm (DV) for the NAc. The aDLS was exclusively targeted here (see Supplementary Fig. S2), as well as in the striatal tissues DA quantification experiment, because compulsive alcohol seeking in rats has recently been specifically associated with this territory (5). After implantation, probes were equilibrated for 1 h with artificial cerebro-spinal fluid (NaCl 149 mM, KCl 2.8 mM, $MgCl_2$ 1.2 mM, $CaCl_2$ 1.2 mM, and glucose 5.4 mM, pH 7.3) at a flow rate of 1 µl/min. Then, dialysis fractions were collected with a refrigerated autosampler (820 Microsampler, Univentor, Zejton, Malta) every 45 min, over a 6-h period divided into a 1h30 period without treatment and a 4h30 period following the injections. At the end of the experiment, the skin was stitched, disinfected, and the rat placed in a heated wake-up cage,

before being replaced in its home-cage after complete awakening. At least one week after the experiment, microdialysis was performed a second time by reversing the dialyzed structures on each hemisphere. After histological validation, DA contents of dialysis fractions were determined using high-performance liquid chromatography (1200 series, AGILENT Technologies, USA), with electrochemical detection (Dionex UltiMate 3000, Thermo Scientific, Illkirch, France) and an Aquasil C18 reverse-phase microcolumn (RP-18, 100 x 2.1 mm, 3 µ particle size, Thermo Scientific, Illkirch, France) maintained at 24°C. The mobile phase (NaH_2PO_4 50 mM, EDTA 0.1 mM, sodium octyl sulfate 1.7 mM, KCl 4.5mM and 5% acetonitrile (vol/vol), adjusted to pH 3.1) was run at a flow rate of 0.4 ml/min. The working electrode potential was +550 mV and the detector sensitivity 2 nM for DA. The running time for each determination was 12 minutes. Chromatograms were collected and treated with integration software (Chromeleon 7 CDS Software, Thermo Scientific). DA concentrations were determined by comparing DA peaks with external standards and were expressed in nanomoles/L.

Behavioral procedures

Intermittent access to 20% alcohol two-bottle choice drinking procedure (IA 20%-EtOH 2BC): This procedure was used to induce escalation of alcohol intake though repeated cycles of intoxication and withdrawal. This procedure leads to a high level of alcohol consumption (See Results Fig. 1B, Fig. 3C, Fig. 5B and Supplementary Fig. S1C) and blood alcohol concentration (≃ 80 mg% after 30 min of drinking), highly relevant to model excessive alcohol use and some signs of dependence (reviewed in (6)). In this study, concurrent access was given on Sundays, Tuesdays, and Thursdays. The placement (left or right) of each solution was alternated between each session to control for side preference, and a bottle of water was placed in a cage without rats to evaluate the spillage that was always ≤ 1 mL (< 3.5% of the total fluid intake). The levels of alcohol and water consumption (g/kg/time) were measured after the first

30 min, allowing investigation of binge-related drinking behavior (*18*), and at the end of the 24h-session.

2.5% Sucrose two-bottle choice drinking procedure: Rats were given 1 h concurrent access to one bottle of water and one bottle of 2.5% (v/v) sucrose. At the end of the session, the volumes of sucrose solution and water consumed were measured to determine sucrose, water and total fluid intake (mL/kg), and preference for sucrose over water (sucrose intake/total intake, expressed as a percentage).

Open arena test: Rats were placed in an opaque open arena apparatus (50 x 50 x 40 cm). The horizontal distance traveled (cm) was recorded over a 30-min period and analyzed with a video-tracking system.

Stepping test: Rats, held by the posterior third of their body, were moved over a length of 90 cm by a rectilinear and regular movement from left to right and inversely along a smooth-surfaced table (7). The test was carried out in triplicate and the number of adjustments of right and left paws during displacement was counted by two observers blind to the experimental conditions.

Light / Dark avoidance test: The apparatus was composed of a light (50 x 40 x 40 cm) and a dark chamber (24 x 40 x 40 cm) separated by an opaque wall with a small aperture (14.5 x 7 cm) allowing rats to move freely between the chambers. The light chamber was opened at the top and lit with a white incandescent light (> 400 lux) located 70 cm above the floor of the chamber. By contrast, the dark chamber was closed at the top and was unlit (< 5 lux). Rats were placed in the center of the light chamber and the total time spent in the light and the dark chambers, over a 5-min period, were recorded with a video-tracking system and analyzed by an observer blind to the experimental conditions.

Elevated plus-maze test: The elevated plus-maze was composed of two opposing open arms (50 x 10 cm) and two opposing arms enclosed by high opaque walls (50 x 10 x 40 cm) suspended 55 cm above the floor. Rats were placed in the center of the elevated plus-maze and times spent in the open and closed arms, over a 5-min period, were recorded and analyzed with a video-tracking system.

Forced swim test: Rats were placed in a transparent cylinder 40 cm high and 20 cm in diameter, filled with water (24 ± 1 °C) to a depth of 30 cm, for 10 minutes (7). Animal activity was recorded and analyzed with a video-tracking system.

Histological analysis

Brain tissue preparation and processing: For striatal tissue DA quantification, rats were deeply anesthetized by isoflurane saturation and euthanized by decapitation. Brains were immediately frozen in liquid nitrogen and stored at -80 °C. For the other experiments, rats were deeply anesthetized by isoflurane saturation or by injection of exagon (Axience SAS, Pantin, France) and transcardially perfused with 0.9% saline followed by 4% paraformaldehyde (PFA) in phosphate-buffered saline (PBS). They were then cryoprotected in 20% sucrose/PB for 24 h and frozen in isopentane cooled to -50°C on dry ice. Coronal sections of striatum and mesencephalon were cut using a cryostat (HM525, Microm, Francheville, France). For striatal tissue DA quantification, aDLS, DMS and NAc were dissected from multiple thick sections of striatum (100 µm, 2.2 to 0.7 mm anterior to bregma) and stored at -80 °C before being homogenized. To verify the placement of the microinjection cannula and microdialysis probes, brains were cut at a thickness of 30 µm. Sections were then stained using Cresyl violet staining and visualized under a light microscope (Nikon Eclipse 80i, TRIBVN, Châtillon, France) coupled to the ICS FrameWork computerized image analysis system (Calopix 2.9.2 software,

TRIBVN, Châtillon, France). Finally, to quantify transgene expression, floating coronal sections (30 µm) of three levels of the mesencephalon were selected according to (1).

Quantification of transgene expression: Free-floating coronal sections were washed with Tris-Buffered-Saline (TBS) and incubated for 1 h in 0.3% Triton X-100 in TBS (TBST) and 3% normal goat serum (NGS). They were then incubated with primary monoclonal mouse anti-TH antibody (1:2500, Millipore catalogue no. MAB5280, RRID:AB_2201526) diluted in TBST containing 1% NGS overnight (4℃). Slices were then incubated with a green fluorescent conjugated goat anti-mouse Alexa 488 antibody (1:500, Invitrogen catalogue no. A-11029, RRID:AB_138404) for 1h30 at room temperature. They were finally mounted on superfrost glass slides (Thermo Scientific, Illkirch, France), with Aqua-Poly/Mount (Polysciences Inc., Hirschberg an der Bergstraße, Germany). Fluorescent acquisitions of TH labelling and mCherry expression were taken with a x20 / NA 0.8 objective on a slide scanner (Axioscan Z1, Zeiss, Göttingen, Germany), and analyzed with ImageJ. Fluorescent illustrations were taken with a x20 / NA 0.8 objective on a spinning-disk confocal microscope (CSU-W1 confocal scanner unit Yokogawa (Gataca Systems, Massy, France), Prime 95B sCMOS Camera (Teledyne Photometrics, Birmingham, UK) and microscope Axio-observer Z1(Zeiss)). Z-stacks of digital images were captured using ZEN software (Zeiss).

Statistical analyses

The sample sizes used in our experiments were estimated based on our previous experience, where power analyses were performed, and the literature in the present fields of investigation. Rats were pseudo-randomly allocated to the different experimental conditions, in order to control for similar basal behavioral performances. Parametric analyses were performed with assumptions of normality (Shapiro-Wilk and Kolmogorov-Smirnov tests). For sphericity, tests were corrected using the Greenhouse-Geisser correction (8). Data were analyzed by two-

sided unpaired t-test, simple linear regression, one-way ANOVA, two-way ANOVAs and RM two-way ANOVAs, three-way ANOVAs, RM three-way ANOVAs depending on the experimental design, using Prism 9 (GraphPad Prism). Due to technical reasons, such as leakage or artefacts obtained in HPLC, some (< 2%) values were missing in the microdialysis experiment. Similarly, during the OSU experiment, a few (< 1%) values were missed du to behavioral recording problems. In these cases, data were analyzed by fitting a mixed model proposed by the statistical software. This mixed model uses a compound symmetry covariance matrix and is fit using Restricted Maximum Likelihood (REML). When indicated, *post hoc* analyses were carried out with Bonferroni's correction procedure. Significance for p values was set at $\alpha = 0.05$. Effect sizes for the ANOVAs were also reported using partial η^2 values (1,9). Determining these values from the mixed-model analysis was however not accessible. Hartigan's test K-means clustering method was performed using JAMOVI software (The Jamovi Project, (2020), open source) to statistically validate the cut-off method for identifying footshock-resistant and footshock-sensitive rats during punished alcohol self-administration. This clustering method allows the classification of homogeneous groups of individuals in a data set, where each cluster is represented by its center, corresponding to the mean of points assigned to the cluster. Thus, individuals classified within the same cluster are considered as similar as possible between them and with the associated center-point. As our population of footshock-resistant and footshock-sensitive rats has a bimodal distribution, we *a priori* defined a minimal *n* cluster = 2. Only one rat, with performances very close to the cut-off, was not classified in the same group using the two methods (Supplemental Fig. 2A) without any change in the results obtained (Supplemental Fig. 2B).

References:

1. Goutaudier R, Coizet V, Carcenac C, Carnicella S. Compound 21, a two-edged sword with both DREADD-selective and off-target outcomes in rats. PLoS ONE. 2020;15(9 September):1–11.
2. Krashes MJ, Koda S, Ye CP, Rogan SC, Adams AC, Cusher DS, et al. Rapid, reversible activation of AgRP neurons drives feeding behavior in mice. Journal of Clinical Investigation. 2011;121(4):1424–8.
3. Paxinos G, Watson C. The Rat Brain in Stereotaxic Coordinates Sixth Edition. Elsevier Academic Press. 2007;
4. Brodnik ZD, España RA. Dopamine uptake dynamics are preserved under isoflurane anesthesia. Neuroscience Letters. 2015 Oct;606:129–34.
5. Giuliano C, Belin D, Everitt BJ. Compulsive alcohol seeking results from a failure to disengage dorsolateral striatal control over behavior. Journal of Neuroscience. 2019;39(9):1744–54.
6. Carnicella S, Ron D, Barak S. Intermittent ethanol access schedule in rats as a preclinical model of alcohol abuse. Alcohol [Internet]. 2014 May;48(3):243–52. Available from: https://linkinghub.elsevier.com/retrieve/pii/S0741832914000494
7. Drui G, Carnicella S, Carcenac C, Favier M, Bertrand A, Boulet S, et al. Loss of dopaminergic nigrostriatal neurons accounts for the motivational and affective deficits in Parkinson's disease. Molecular Psychiatry [Internet]. 2014 Mar 12;19(3):358–67. Available from: http://www.nature.com/articles/mp20133
8. Abdi H. Greenhouse–Geisser Correction. In: Salkind N, editor. Encyclopedia of Research Design [Internet]. 2455 Teller Road, Thousand Oaks California 91320 United States : SAGE Publications, Inc.; Available from: http://methods.sagepub.com/reference/encyc-of-research-design/n168.xml
9. Levine TR. Eta Squared, Partial Eta Squared, and Misreporting of Effect Size in Communication Research. Human Communication Research [Internet]. 2002 Oct 1;28(4):612–25. Available from: http://doi.wiley.com/10.1093/hcr/28.4.612

SUPPLEMENTAL FIGURES

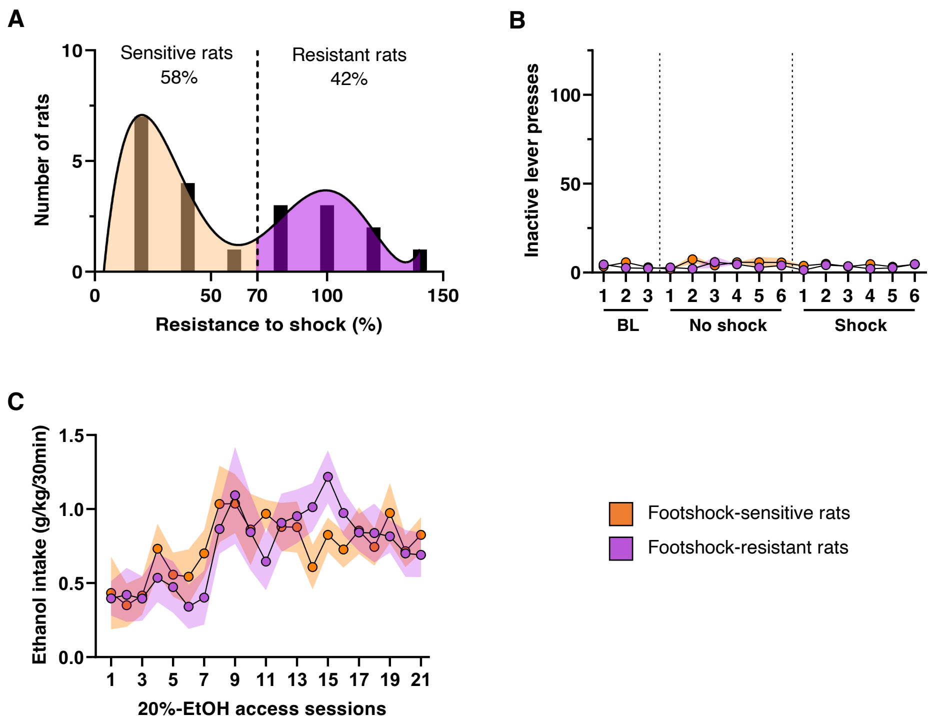

Supplementary Fig. S1. Behavioral characterization of footshock-sensitive and footshock-resistant rats. (A) Bimodal distribution of the population of rats self-administering alcohol under footshock-punishment sessions. 42% of rats were *footshock-resistant* (n = 9) and 58% were *footshock-sensitive* (n = 12). **(B)** Number of inactive lever presses in 30-min self-administration sessions of 20% EtOH (FR3), during baseline (BL), "no-shock" and "shock" sessions. RM two-way ANOVAs found no effect of treatment, transgene or treatment x transgene interaction [$F_s < 1.06$, $P > 0.39$, partial $\eta^2 < 0.05$]. **(C)** Ethanol intake during the first 30 minutes of IA 20%-EtOH 2BC. RM two-way ANOVAs showed a significant effect of session [$F_{(8, 158)} = 3.41$, $P < 0.001$, partial $\eta^2 = 0.15$) but neither effect of group nor session x group interaction [$F_s < 0.72$, $P > 0.5$, partial $\eta^2 < 0.04$]. Data are expressed as means ± SEM.

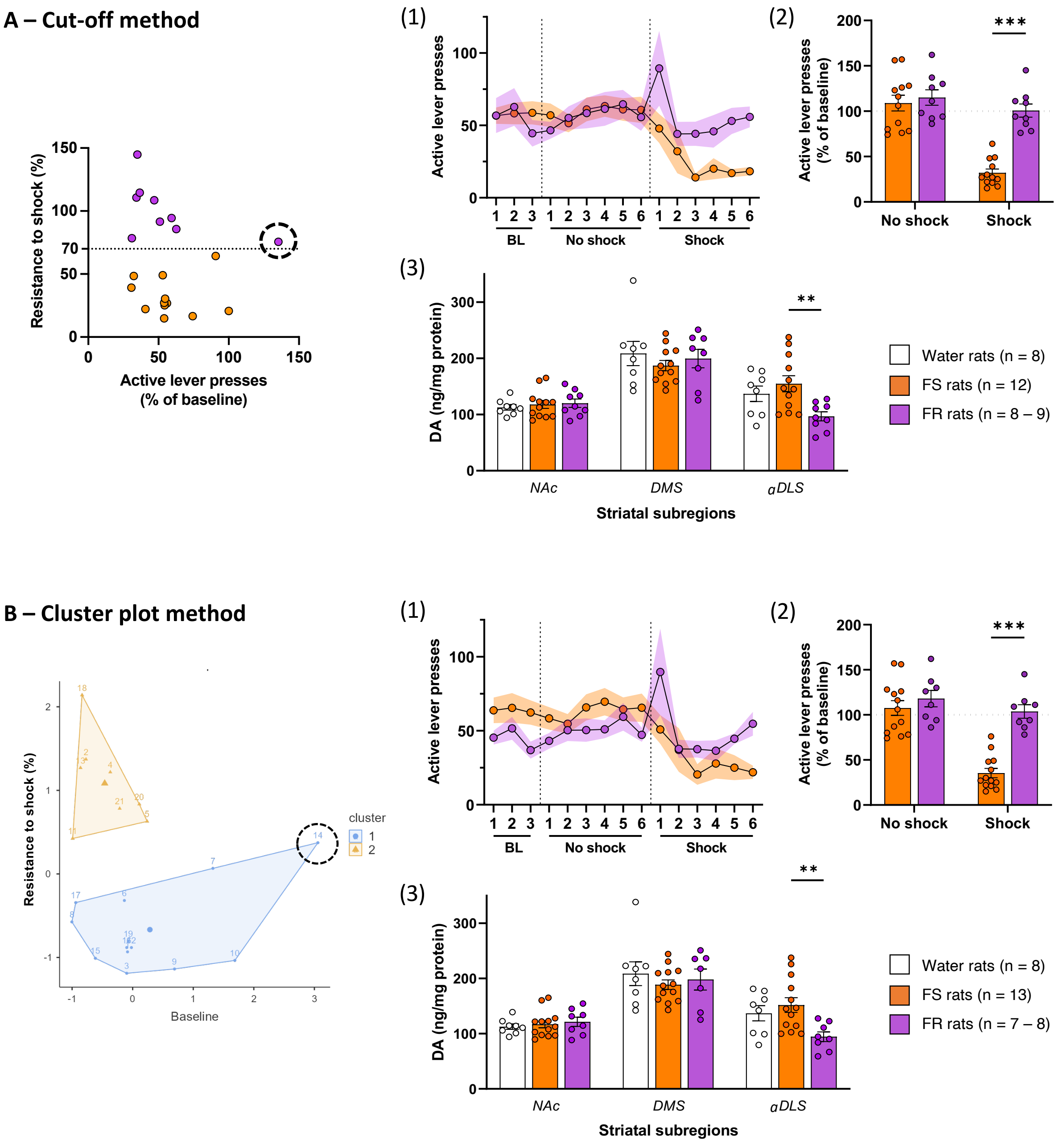

Supplementary Fig. S2. Clustering of data using the "cut-off" (A) or "cluster plot" (B) method. The dotted circle indicates the unique rat that diverges between these two methods. In panel A and B, (1): number of active lever presses in 30-min self-administration sessions of 20% EtOH (FR3), during baseline (BL), "no-shock" and "shock" sessions in *footshock-sensitive* (FS) and *footshock-resistant* (FR) rats. RM two-way ANOVA showed a significant group x session interaction [**(A)** $F_{(14, 266)} = 2.86$, $P < 0.001$, partial $\eta^2 = 0.13$ vs **(B)** $F_{(14, 266)} = 3.32$, $P < 0.001$, partial $\eta^2 = 0.15$]. In panel A and B, (2): mean active lever presses during the

last three “no-shock” sessions and the last three “shock” sessions normalized to baseline. RM two-way ANOVA showed a significant shock condition x group interaction [**(A)** $F_{(1, 19)} = 20.89$, $P < 0.001$, partial $\eta^2 = 0.52$ vs **(B)** $F_{(1, 19)} = 14.59$, $P < 0.001$, partial $\eta^2 = 0.43$]. In panel A and B, (3): NAc, DMS and aDLS DA levels for FR, FS and Water rats. RM mixed-effects model showed a significant group x striatal subregion interaction [**(A)** $F_{(4, 77)} = 2.96$, $P < 0.05$ vs **(B)** $F_{(4, 77)} = 2.68$, $P < 0.05$]. Data are expressed as mean ± SEM. Bonferroni correction post-hoc analysis: **, $P < 0.01$; ***, $P < 0.001$.

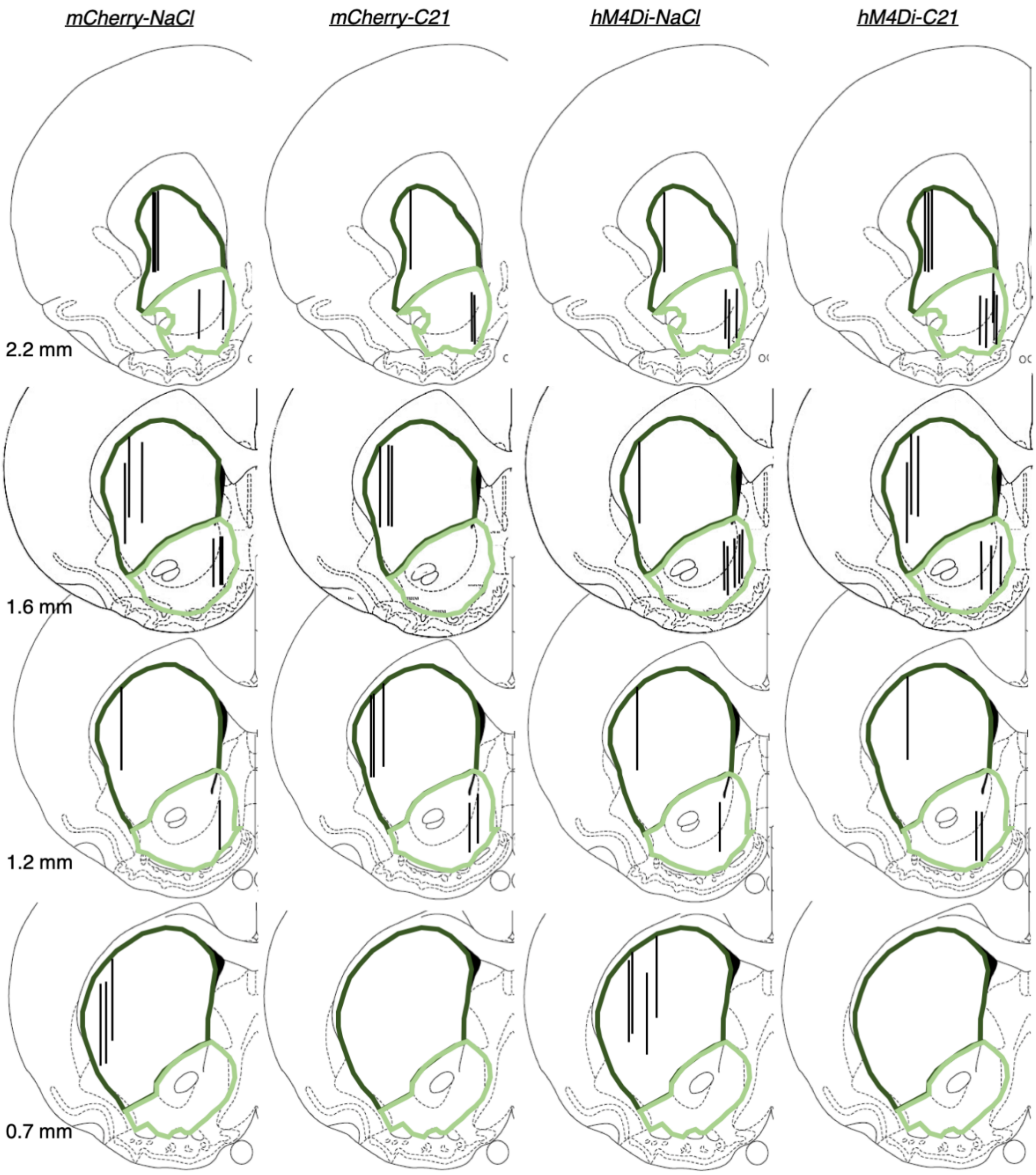

Supplementary Fig. S3. Schematic representation of the microdialysis probe placement across groups. The location of the dialysis membrane in each individual is represented by vertical bars. The dorsal striatum and the nucleus accumbens are delineated in dark and light green respectively. The numbers indicate the distance anterior to bregma (mm). Coronal sections were taken from (3).

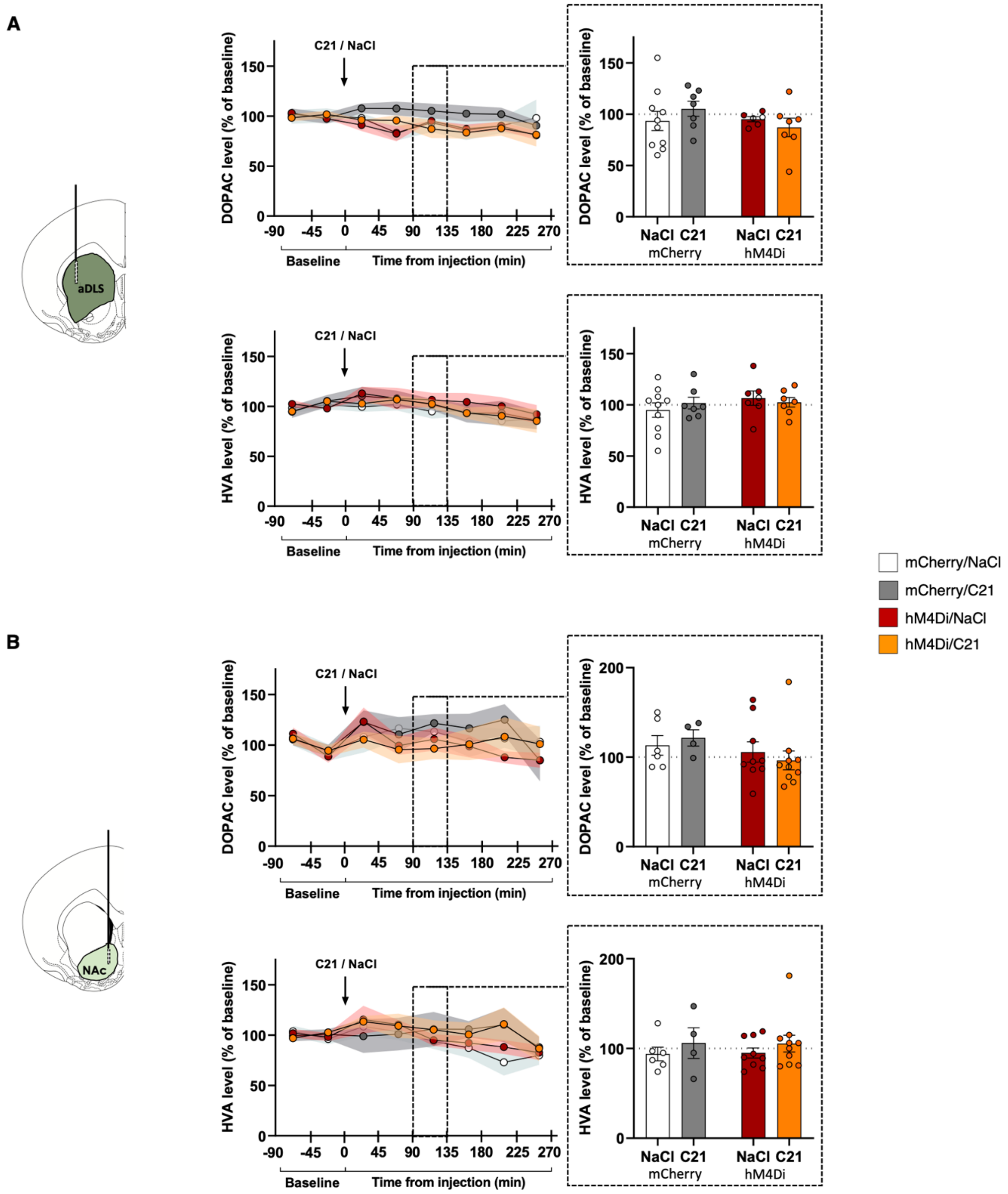

Supplementary Fig. S4. Chemogenetically-induced nigrostriatal hypodopaminergia does not change DA turn-over rate. (A - B) Extracellular DOPAC and HVA concentrations in aDLS (A) and NAc (B) in eight 45 min-fractions collected by microdialysis. Data are normalized to baseline. **(A)** Time course of extracellular DOPAC and HVA concentrations in the aDLS of *hM4Di* rats treated with C21 (orange, $n = 7$) or NaCl (red, $n = 6 - 7$) and *mCherry* rats treated with C21 (grey, $n = 7$) or NaCl (white, $n = 10$). **(B)** Time course of extracellular

DOPAC and HVA concentrations in the NAc of *hM4Di* rats treated with C21 (orange, $n = 10$) or NaCl (red, $n = 9$) and *mCherry* rats treated with C21 (grey, $n = 4$) or NaCl (white, $n = 6$). In the fraction collected between 90 and 135 minutes after injection (dotted squares), two-way ANOVA revealed no effect of the transgene, treatment nor treatment x transgene interaction in the aDLS or in the NAc [$F_s < 1.77$, $P > 0.2$, partial $\eta^2 < 0.07$]. Data are expressed as mean ± SEM.

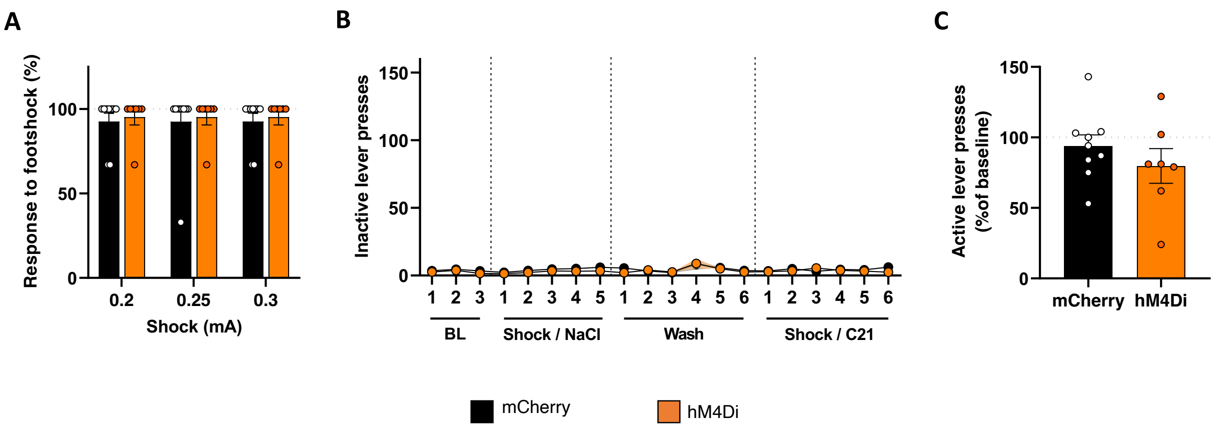

Supplementary Fig. S5. Chemogenetically-induced nigrostriatal hypodopaminergia does not decrease sensitivity to footshock and does not increase inactive lever presses or operant ethanol self-administration in absence of footshock. (A) In order to ensure that chemogenetic did not decrease sensitivity to footshock, we evaluate the effect of C21 on the percentage of response to footshock in absence of alcohol, at intensity used during self-administration of 20%-EtOH (0.25 mA) and at two surrounding intensities (0.2 and 0.3 mA) in *hM4Di* rats (orange, $n = 7$) and *mCherry* rats (black, $n = 10$). RM two-way ANOVAs found no effect of the group, intensity or group intensity interaction [$F_s < 0.41$, $P > 0.5$, partial $\eta^2 < 0.01$]. **(B)** Number of inactive lever presses in 30-min self-administration sessions of 20%-EtOH (FR3), during baseline (BL), "Shock/NaCl", "Wash" and "Shock/C21" sessions. RM two-way ANOVA showed no effect of session, transgene or session x transgene interaction [$F_s < 1.95$, $P > 0.1$, partial $\eta^2 < 0.12$]. **(C)** Effect of C21 on the percentage of lever presses over the course of a 30-min self-administration session of 20%-EtOH (FR3) without shock normalized to baseline. Unpaired t-test showed no difference of performances between the two groups [$t = 0.98$, $P = 0.34$]. Data are expressed as mean ± SEM.

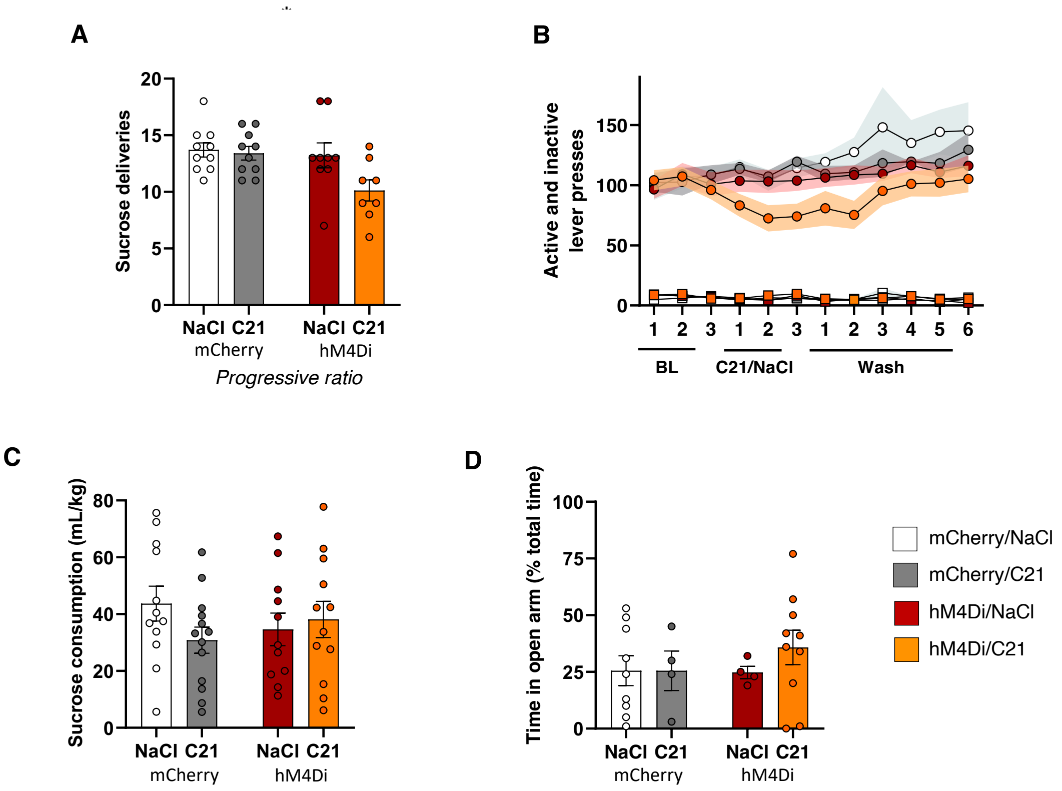

Supplementary Fig. S6. Nigrostriatal hypodopaminergia does not altered reward sensitivity or induce anxiety-related behavior. (A) Number of 2.5%-sucrose deliveries obtained in 60-min self-administration (SA) sessions under a progressive ratio of reinforcement, in *hM4Di* rats treated with C21 (orange, $n = 8$) or NaCl (red, $n = 9$) and *mCherry* rats treated with C21 (grey, $n = 10$) or NaCl (white, $n = 10$). Two-way ANOVA showed a significant effect of transgene and treatment [$F_s > 4.26$, $P < 0.05$, partial $\eta^2 > 0.11$], but only a marginal transgene x treatment interaction [$F_{(1, 33)} = 2.89$, $P = 0.099$, partial $\eta^2 = 0.08$]. **(B)** Number of active (circle) and inactive (square) lever presses to obtained 2.5%-sucrose deliveries in 60-min self-administration sessions under continuous reinforcement (FR1), during baseline (BL), "C21/NaCl" and "Wash" sessions, in *hM4Di* rats treated with C21 (orange, $n = 8$) or NaCl (red, $n = 9$) and *mCherry* rats treated with C21 (grey, $n = 10$) or NaCl (white, $n = 10$). On active lever presses, RM three-way ANOVAs found a significant effect of session and transgene [$F_s > 4.01$, $P < 0.05$, partial $\eta^2 > 0.11$] but no effect of treatment or any interaction

between these factors [$F_s < 2.42$; $P > 0.1$; partial $\eta^2 < 0.06$]. On inactive lever presses, RM three-way ANOVAs revealed a significant effect of session [$F_{(7, 218)} = 2.65$; $P < 0.05$; partial $\eta^2 < 0.07$] but no effect of treatment, transgene, or any interaction between these factors [$F_s < 1.14$; $P > 0.33$; partial $\eta^2 < 0.33$]. **(C)** Sucrose consumption during a 60-min 2.5%-sucrose two-bottle-choice drinking session by *hM4Di* rats treated with C21 (orange, *n* = 12) or NaCl (red, *n* = 11) and *mCherry* rats treated with C21 (grey, *n* = 13) or NaCl (white, *n* = 12). **(D)** Percentage of time spent in open arms of an elevated-plus maze by *hM4Di* rats treated with C21 (orange, *n*=10) or NaCl (red, *n*=4) and *mCherry* rats treated with C21 (grey, *n* = 4) or NaCl (white, *n* = 9). Two-way ANOVAs showed no effect of treatment, transgene or treatment x transgene interaction in these two tests [$F_s < 2.04$; $P > 0.16$; partial $\eta^2 < 0.04$]. Data are expressed as mean ± SEM.

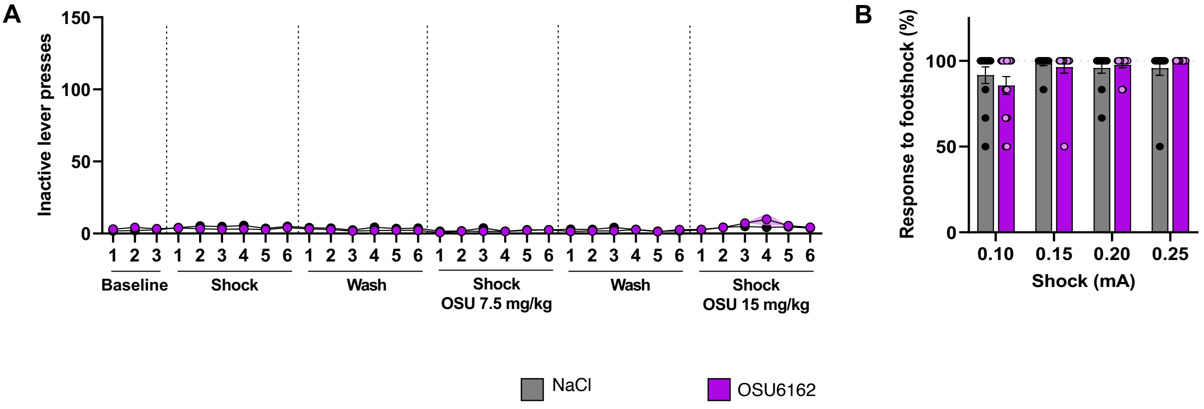

Supplementary Fig. S7. OSU6162 does not increase inactive lever presses during punished operant ethanol self-administration or footshock sensitivity. (A) Number of inactive lever presses in 30-min self-administration sessions of 20%-EtOH (FR3), during baseline, “Shock”, “Wash” and “Shock OSU 7.5 mg/kg” or “Shock OSU 15 mg/kg” sessions. Mixed-effect model analysis showed a significant effect of session [$F_{(32,761)} = 2.63$, $P < 0.001$], but no effects of treatment or session x treatment interaction [$F_s < 1.05$, $P > 0.4$]. **(B)** To ensure that OSU6162 at 15 mg/kg compound did not increase sensitivity to footshock, we evaluate the effect of OSU6162 on the percentage of response to footshock in absence of alcohol, at intensity used during punished self-administration of 20%-EtOH (0.25 mA) and at three lower intensities (0.1; 0.15 and 0.2 mA) in OSU rats (purple, *n* = 14) and NaCl rats (grey, *n* = 12). RM two-way ANOVAs found a significant intensity effect [$F_{(1, 41)} = 3.53$, $P < 0.05$, partial $\eta^2 = 0.13$], but no effect of treatment, nor treatment x intensity interaction [$F_s < 0.91$, $P > 0.44$, partial $\eta^2 < 0.04$]. Data are expressed as mean ± SEM.

Table S1. Summary of statistical analyses
